# Bibliography

**Published:** 1901-05-15

**Authors:** 


### BIBLIOGRAPHY.

"Poems of the Farm," a collection of poems from the pen of Dr. Charles Nelson Johnson, of Chicago, has just appeared from the Daniels Company Press, Chicago. In a preferatory note the author disclaims any idea of literary merit, and states that they are not intended for the general public, but are printed at the urgent request of his friends who have become familiar with them as they have appeared from time to time in various publications. Now, we do not profess to be a literary connoisseur, especially in poetical art; we haven't any competent value of either rhyme or measure, and consequently feel utterly incapable of passing a literary criticism upon these verses. But we picked up the little book and began to read and could not put it down until we had read it all. Then we read it aloud to our good wife, who is both literary, musical and a fair judge of poetry, and she concluded that it was not only beautiful, but good, and that it touched in a most delightful manner some of the better sentiments which ought to predominate in the home life. No one can read such sentiments without being inspired to nobler living, and Dr. Johnson would have done wrong not to have given his verses to the public. The title of the little book does not convey any adequate idea of its contents, as some of the choicest verses are devoted to the nursery life in a great city.

